# Identifying and Prioritizing Greater Sage-Grouse Nesting and Brood-Rearing Habitat for Conservation in Human-Modified Landscapes

**DOI:** 10.1371/journal.pone.0026273

**Published:** 2011-10-13

**Authors:** Matthew R. Dzialak, Chad V. Olson, Seth M. Harju, Stephen L. Webb, James P. Mudd, Jeffrey B. Winstead, L.D. Hayden-Wing

**Affiliations:** Hayden-Wing Associates LLC, Natural Resource Consultants, Laramie, Wyoming, United States of America; University of Western Ontario, Canada

## Abstract

**Background:**

Balancing animal conservation and human use of the landscape is an ongoing scientific and practical challenge throughout the world. We investigated reproductive success in female greater sage-grouse (*Centrocercus urophasianus*) relative to seasonal patterns of resource selection, with the larger goal of developing a spatially-explicit framework for managing human activity and sage-grouse conservation at the landscape level.

**Methodology/Principal Findings:**

We integrated field-observation, Global Positioning Systems telemetry, and statistical modeling to quantify the spatial pattern of occurrence and risk during nesting and brood-rearing. We linked occurrence and risk models to provide spatially-explicit indices of habitat-performance relationships. As part of the analysis, we offer novel biological information on resource selection during egg-laying, incubation, and night. The spatial pattern of occurrence during all reproductive phases was driven largely by selection or avoidance of terrain features and vegetation, with little variation explained by anthropogenic features. Specifically, sage-grouse consistently avoided rough terrain, selected for moderate shrub cover at the patch level (within 90 m^2^), and selected for mesic habitat in mid and late brood-rearing phases. In contrast, risk of nest and brood failure was structured by proximity to anthropogenic features including natural gas wells and human-created mesic areas, as well as vegetation features such as shrub cover.

**Conclusions/Significance:**

Risk in this and perhaps other human-modified landscapes is a top-down (*i.e.*, human-mediated) process that would most effectively be minimized by developing a better understanding of specific mechanisms (*e.g.*, predator subsidization) driving observed patterns, and using habitat-performance indices such as those developed herein for spatially-explicit guidance of conservation intervention. Working under the hypothesis that industrial activity structures risk by enhancing predator abundance or effectiveness, we offer specific recommendations for maintaining high-performance habitat and reducing low-performance habitat, particularly relative to the nesting phase, by managing key high-risk anthropogenic features such as industrial infrastructure and water developments.

## Introduction

A promising approach to animal conservation in human-modified landscapes involves quantifying resource-related behavior that structures occurrence, linking occurrence with a demographic outcome such as reproductive success, and depicting spatially the coincidence of occurrence and successful demographic performance at the landscape level [1]. Making the link between resources and fitness is necessary for guidance of on-the-ground conservation action because it distinguishes higher-risk habitat where animals are likely to occur, yet have poor demographic performance, from lower-risk habitat in which occurrence coincides with successful demographic performance. This approach has strong application in conservation planning where wildlife-human interaction is a concern because it offers a spatially explicit framework from which to identify critical habitat [2], and the analysis underpinning spatial predictions provides information on mechanisms driving observed spatial or demographic patterns. Prioritizing conservation action based on occurrence alone, without a connection to fitness, risks channeling conservation effort and funding into an area that essentially functions as a demographic sink [3], [4], [5]. Research linking resource selection with fitness remains relatively uncommon [1], [6], [7]; less common still is research that makes this link relative to influences of human activity on discrete critical life-history phases in animals such as season-specific survival or rearing of young [2].

A conservation issue of emerging interest in human-modified landscapes is the influence of human activity on the relationship between predators and prey [8]. One important way that humans influence this relationship is through the unintended provision of resource subsidies such as food or refugia [9], [10]. Resource subsidies established through human activity such as agriculture, urbanization, or industrial development have been shown to alter the interaction between predators and prey by modifying predator or prey abundance, or by enhancing predator effectiveness in ways that have behavioral or demographic consequences in prey populations [8], [9], [10], [11].

In portions of western North America, human activity associated with development of energy resources is of broad conservation interest because its presence has increased in recent decades along with concern about its potential impact on wildlife habitat and the interaction between predators and sensitive prey species [12], [13]. A good example of this increase can be seen in Wyoming, USA where the number of producing wells in the 25 largest natural gas fields in the state increased from 7,907 wells in 2000 to 35,821 wells in 2008 [14]. One species in particular, the greater sage-grouse (*Centrocercus urophasianus*; hereafter, sage-grouse), has brought into focus the challenges associated with balancing wildlife conservation with increasing demand for energy resources. The sage-grouse is distributed throughout shrub-steppe habitat in 11 American states and 2 Canadian provinces and is strongly associated with several species of sagebrush (*Artemisia* spp.). Long-term population declines of 17–47% have been observed throughout much of the species distribution [15], [16]. In 2010, listing of sage-grouse as federally threatened or endangered within the U.S. was found to be warranted under the Endangered Species Act, but listing was precluded by higher priority listing actions [17].

Among factors that are associated with decline in sage-grouse populations, such as large-scale changes in temperature and precipitation cycles, fire, and predation [15], [18], only one factor–human activity–is readily amenable to conservation intervention. Human activity associated with energy development has been linked to changes in resource use, decline in attendance and activity at breeding locations, and decline in survival [19], [20], [21], [22]. Importantly, many of the largest energy reserves in western North America coincide with shrub-steppe habitat, occurrence of sage-grouse, and public land where federal authorization of development has increased pursuant to initiatives to reduce dependency on over-seas energy sources [23], [24]. Common ground shared among state government and wildlife agencies, and industry is the aim to prevent further decline in sage-grouse and retain local (*i.e.*, state) administrative authority over its management. In Wyoming, the core of the current distribution of sage-grouse, large-scale efforts have been undertaken to identify landscapes that are most important in sage-grouse population persistence, to quantify specifically how energy development affects populations, and to develop strategies by which development may be balanced with long-term population viability [25], [26], [27], [28]. Still needed is a series of regional landscape assessments of how specific landscape features are important to sage-grouse (*sensu* [29]) during critical life history phases such as reproduction, winter, and migration, and spatially explicit guidance for avoiding or minimizing impact as part of development projects for which the interaction between humans and sage-grouse is unavoidable.

In this paper, we relate reproductive success in female sage-grouse fitted with solar powered Global Positioning Systems (GPS) units to seasonal patterns of resource selection, with the larger goal of developing a spatially-explicit framework for managing human activity and sage-grouse conservation at the landscape level. First, we estimated resource selection functions (RSFs; [30]) to quantify the spatial pattern of nest occurrence, resources selected by females during incubation, and resources selected by females during brood-rearing (including night-time locations). Second, we estimated Cox proportional hazards models [31] to quantify risk of nest and brood failure, and we mapped probability of nest and brood failure at the landscape level. Third, we combined spatial models of occurrence and risk to provide a spatially-explicit assessment of the relationship between habitat and demographic performance, wherein low-performance habitat was defined as high probability of occurrence coupled with high risk of nest or brood failure, and high-performance habitat was high probability of occurrence coupled with low risk of nest or brood failure (*sensu* [1]).

## Materials and Methods

### Study Area

The 5,625 km^2^ study area included portions of the Wind River Basin in central Wyoming, USA. Topography is variable with gently sloping flats, dry washes, and rocky canyons ranging in elevation from 1,478–2,776 m. Average maximum and minimum temperature during the study period (April–August) was 23.9 and 5.4^°^C; average precipitation during the study period was 2.7 cm (C.V. Olson, unpublished data). Dominant plant species at lower elevation included Wyoming big sagebrush (*Artemisia tridentata wyomingensis*), basin big sagebrush (*A. t. tridentata*), black greasewood (*Sarcobatus vermiculatus*), and shadscale (*Atriplex confertifolia*). At higher elevation, mountain big sagebrush (*A. t. vaseyana*), limber pine (*Pinus flexilis*), and rocky mountain juniper (*Juniperus scopulorum*) were present. Predators of sage-grouse (including nests) included common raven (*Corvus corax*), golden eagle (*Aquila chrysaetos*), coyote, (*Canis latrans*), and American badger (*Taxidea taxus*). The study area encompassed historic and ongoing ranching and development of energy resources. Oil and natural gas development was initiated in the 1920s; gas development accelerated in the 1990s. In 2010 there were 1,085 wells associated with oil and gas development in the study area. Among the notable features of human activity is the creation of mesic habitat throughout otherwise xeric (lower elevation) portions of the study area. Water produced as a byproduct of energy development, along with agricultural developments such as stock ponds, established the presence of low-elevation mesic habitat in the study area.

### Field Procedures, Location and Mortality Data

From 2008–2010, we captured female sage-grouse by spotlighting [32] during spring (generally March and April) on and around leks that were dispersed throughout the study area. Some capture occurred in autumn (generally September through November). We determined age and sex [33], and fitted sage-grouse with 30-g ARGOS/GPS Solar PTTs (PTT–100, Microwave Telemetry Inc., Columbia, MD 21045 USA) using a rump-mount technique. GPS units were configured with Ultra High Frequency (UHF) beacons for ground tracking and detection of fatality and had a 3-year operational life. Collars were programmed to record location information from 0700–2200 every 3 h during 16 Feb–14 May, and every 1 hr during 15 May–15 July. During 16 July–1 September collars recorded location information every 3 hr from 1000–2200. Animal capture and handling protocols were approved by the Wyoming Game and Fish Department (Chapter 33 Permit #649).

Nests were checked immediately after females departed the area to confirm nest fate. Nests were considered successful if females incubated for ≥24 days and a ground visit verified≥1 egg hatched. Hatched eggs were identified by the presence of an egg cap and an intact egg membrane (initial cracking, or pipping, of the egg typically results in two eggshell fragments, with the smaller fragment called the cap). Such features are not typical of depredated eggs. Nests were classified as unsuccessful if females vacated the nest>3 days before the expected hatch date and a ground visit confirmed nest failure. Upon visitation, depredation as a cause of nest failure was assigned to a nest when eggs had been removed or when eggshell fragments indicated destruction of the egg in a manner consistent with observed depredation patterns [34], [35], [36], [37], [38]. Brood surveys were conducted weekly beginning when chicks were about 7 days old. Efforts were made to determine brood status (presence versus absence of a brood) without flushing females. The presence of chicks was determined by observation and, in some cases, based on behavior of the female (i.e., the female walking or running away from the observer rather than flying, becoming defensive or aggressive, or displaying wing-dragging or flutter-hopping behavior [39]). Broods were classified as failed if the female flew long distances (*i.e.*, steplength ≥2 km based on GPS data) when the chicks were<10 days old or if no chick was found with the female on two consecutive surveys. Broods were considered successful if ≥1 chick survived to 35 days old. Broods were monitored in the field until mid August or until brooded females aggregated with other birds.

### Covariate Calculation

Using a Geographic Information System (GIS; ArcGIS® 9.3, ESRI, Redlands CA), we calculated covariates depicting landscape features that, based on field observation and previous research, influenced behavior of sage-grouse [2], [19] (Table S1). Raster images of oil and natural gas wells were updated annually so that we could analyze location data against wells known to be present at the time the relocation occurred. Raster images of all other human modifications of the landscape (Table S1) were developed based on 2006 aerial imagery and updated using 2009 imagery. We used Spatial Analyst in ArcGIS® to calculate raster images and to extract values from raster data to location data for all covariates.

### Covariate Inclusion

We used a multi-step covariate inclusion procedure. First, we fitted univariable conditional logistic regression models for all covariates and eliminated from further consideration covariates for which p-values associated with the Wald statistic were>0.2 [40]. Univariable conditional logistic regression was implemented in the PHREG procedure in SAS 9.2® (Cary, NC USA; see below for details). Second, after conducting univariable logistic regressions we assessed correlation among covariates using the CORR procedure in SAS® and eliminated a covariate if correlation with another variable was high (Pearson product-moment correlation ≥|0.7|), retaining the covariate with the lowest Akaike Information Criterion (AIC). At this point (after steps 1 and 2), remaining covariates were considered to comprise “full” models. The final component of the covariate inclusion procedure was multi-variable conditional logistic regression using the PHREG procedure, specifying the SAS® option *selection = stepwise*, which implements a series of alternating forward selection and backward elimination steps to generate “reduced” (final) models. Criteria for covariate entry and retention were p ≤0.1 and p ≤0.05, respectively. Covariate inclusion methods were applied to all resource selection and survival models (see below).

### Resource Selection Modeling-General Approach

We estimated RSFs for 3 components of reproduction in sage-grouse: selection of the nest site, resources selected during egg laying and incubation, and resources selected during brood-rearing. The general approach included a matched design in which each observed location was associated with a specific set of random locations drawn from within a limited spatial domain [41], [42]. A matched design: 1) enables evaluation of used locations relative to random locations that represent true absences (because an animal cannot also be at random locations at the time it occupies its actual location [42]); 2) is a powerful approach when resource availability may change through time (as in human-modified landscapes [43]); and 3) is appropriate when the distance an individual moves between successive relocations is short relative to its entire seasonal range (as is the case with gallinaceous birds with broods [44]). As part of this approach, we estimated RSFs using conditional logistic regression [42], [44], [45], [46], [47].

### Selection of the Nest Site-A Matched Design Based on Age-Specific Availability

To quantify the spatial pattern of nest occurrence, we matched each known nest site in our study area (*n* = 88) with a set of 200 non-used locations and considered each nest location with its associated 200 non-used locations as a single stratum (*sensu* [48]). For second-year birds (those attempting nesting for the first time and thus without a previous nest location to which fidelity may be shown), the set of non-used locations were drawn from within a circular buffer (see below) of the lek at which breeding was suspected. GPS units offered detailed information on lek attendance, including time-of-day, allowing us to estimate with confidence the lek at which breeding occurred. The radius of the buffer centered on the lek at which breeding occurred was defined uniquely for each bird as the distance between the lek and the nest plus 20% of that distance. We enforced a minimum radius equal to the median distance between the lek and the nest across all second-year birds (2,585 m) to acknowledge that, although some birds nested within a short distance from the lek, habitat farther from the lek also was available. The minimum and maximum distance from a lek of breeding at which second-year birds established a nest was 388 m and 9,059 m, respectively. For after-second-year birds (individuals that likely attempted nesting in previous years), non-used locations were drawn from a 2-km circular buffer centered on the actual nest location to acknowledge that availability and selection of the current nest location was constrained by fidelity to the previous year's nesting area [49], [50] (*but see* [51]). Our data show that, among 12 birds that nested in at least 2 consecutive years during the study, the maximum distance between inter-year nesting attempts was 1.7 km. Considering nest-area fidelity further, we evaluated whether a 0-, 1-, or 2-year time lag in response to oil or gas well development was an important predictor of nest occurrence [21], and we included a time-lag by age interaction term.

### Egg-Laying and Incubation, And Brood-Rearing-A Discrete Choice Approach

To quantify resource selection during the period that encompassed egg-laying and incubation, we first eliminated locations that were within 20 m of the nest (assuming that these locations reflected roosting on the nest plus telemetry error). We matched each remaining bird location (reflecting forays or off-bouts) with a set of 3 non-used locations drawn from within a circular buffer centered on the nest. The radius of the buffer was defined uniquely for each bird as the maximum distance moved from the nest during egg-laying or incubation plus 20% of that distance. This design quantifies a choice made by an individual female sage-grouse (*i.e.*, used location) relative to 3 alternative choices that also were available temporally and spatially but were not chosen (*i.e.*, non-used locations). In cases when individuals exhibit central-place behavior, such as during incubation, assuming proportional availability within a given spatial domain may not be reasonable. We expected the probability of resource use to decline with distance from the nest [52] (*also see* [30], [45], [53]) so, in all models of resource selection during egg-laying and incubation, we included the distance term ln(*d*) *e^−d^* ^/100^ where *d* is the distance between each location and the nest, *e* ≈2.718 (base for natural logarithms), and 100 is a decay constant [54], [55]. Also, we expected movement, and hence availability, to change across different times of day so we included a term that interacted time-of-day with the distance term [56]. GPS data provided clear indication of nest initiation, incubation, and hatching/failure. We considered egg-laying to occur during a 12-day period before incubation [57]. Hatching/failure was identified as movement of the female from the nest with no subsequent movement back to the nest, and was confirmed by field visitation to the nest after hatching or failure.

Brood-rearing by sage-grouse in this study area was characterized by a shift from xeric (basin) to more mesic (mountainous) habitat when chicks were 35–40 days old. Given this, and the importance biologists place on thoroughly understanding brood-rearing behavior, we quantified resource selection during the early (0–21 days), mid (22–42 days), and late (>42 days) brood-rearing periods separately, based on the age of chicks. To define resource availability, we first calculated the distance between successive bird locations *l*_0_ and *l*_1_ and tallied this distance into 50-m bins. This established a distribution of observations from which random distances were drawn and associated with each *l*_0_. The area from which locations (non-used) could be drawn as alternative choices to location *l*_1_ was defined as a circular buffer centered on *l*_0_ with a radius equal to a distance randomly assigned from the distribution of observed distances between successive locations [48]. We imposed two constraints on this process. First, the randomly assigned distance was required to be ≥ to the observed distance between *l*_0_ and *l*_1_. Second, we also enforced a minimum distance for each random point equal to the median distance between successive locations observed across all individuals (69 m) to acknowledge there was a minimum area available even if an individual did not move during that time period on a given day. We matched each bird location with a set of 3 non-used locations drawn from within the area of availability and considered each used location with its associated 3 non-used locations as a single stratum. As with the egg-laying and incubation analysis, we included the distance-decay term (from *l*_0_ to *l*_1_ and the 3 associated non-used locations) and the interaction between time-of-day and the distance term. We excluded from analysis bird locations for which the distance between successive locations was in the largest 1% of all distances to remove long distance movements (*i.e.*, movements not related to patch-level selection) and equipment failures (*i.e.*, missing GPS locations).

### RSF Analysis

We estimated fixed-effects RSF models using conditional logistic regression implemented in the PHREG procedure in SAS® specifying Breslow's approximation for likelihood calculation (*ties = Breslow*; [58]). We aimed to estimate RSFs for each reproductive phase separately within each year but because of the small size of some within-year samples we pooled data across years. We thus estimated separate pooled-across-years RSFs for early, mid, and late brood-rearing periods. We natural log-transformed all distance variables (in SAS®, *new* = *log*(*original* +0.1) to allow a functional form of the relationship between resource selection and a distance-based covariate that depicted a decreasing magnitude of influence with increasing distance (adding 0.1 assures that a natural log transformation is not attempted on a cell with value = 0). We developed and evaluated a quadratic term (*quadratic* = *original* ^*^ *original*) for fractional vegetation covariates, elevation, and slope because animals often avoid the lowest and highest values associated with a given landscape feature [2], [59]. When modeling higher-order terms (*i.e.*, quadratic) it is necessary to also include lower-order terms in the model. In the case of modeling a quadratic polynomial, the lower-order term represents the overall effect of the covariate; without including the linear term the covariate effect will be depicted as a monotonically increasing or decreasing parabola with minimum or maximum values at the origin [60]. We created and analyzed interaction terms for which we could see biological relevance; for example, we evaluated whether a significant interaction occurred between anthropogenic features and vegetation or topographic features. We explored resource selection at night during brood-rearing by conducting separate analyses using locations corresponding to 2200 h (but not during incubation because females roosted on the nest).

### Survival

We estimated cumulative survival *S*(*t*) of nests and broods using the Cox proportional hazards model [31] implemented by the PHREG procedure in SAS®. The proportional hazards model is typically expressed as 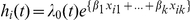
which states that the hazard function *h* for individual *i* at time *t* is the product of an unspecified non-negative baseline hazard function λ_0_(t) and an exponentiated linear function of covariates *k* [61]. In the Cox model, covariate effects are interpreted in terms of hazard ratios (exp[β]). Hazard ratios>1.0 indicate an increasing risk of an event (fatality) with increasing values for the covariate. Hazard ratios<1.0 indicate a decreasing risk of fatality with increasing values for the covariate. Hereafter, we refer to application of the Cox model in terms of risk of nest or brood failure. To estimate risk of nest failure, it was necessary to account for correlated observations within subjects; that is, some females attempted nesting multiple times. Dependence among observations can lead to artifactually declining hazard functions, biased test statistics, and biased measures of precision [61]. To fit a model that accounts for correlated observations within subjects, we first performed the covariate inclusion methods described above, and then used a counting-process style of input [62] that included the SAS® statement *covs*(*aggregate*) coupled with the statement *id* = *individual*. The *covs* option requests a robust sandwich estimate for the covariance matrix resulting in a robust standard error for parameter estimates. The *aggregate* keyword adds residual score components (*i.e.*, *dfbetas*, which provide a measure of influence a particular case has on a coefficient estimate) within the entity specified in the *id* statement before computing the covariance matrix [63], [64]. We used Breslow's approximation for likelihood calculation.

Estimating risk of brood failure introduced several additional considerations. First, this study involved monitoring females via telemetry; no chick was monitored via telemetry. We made the assumption that landscape features encountered by broods were a direct reflection of features encountered by females. In no instance was a brood observed without the attending female. Second, we recorded many relocations on brooded females, yet the structure of time-to-event analysis requires that each record (*i.e.*, a given brood's fate) include a single value for each covariate. To accommodate this, we calculated within-brood adjusted means (least-squares means in SAS®), a measure of precision (SD), and the coefficient of variation (CV) for each covariate using the GLM procedure in SAS®. We performed the covariate inclusion methods described above and we included mean values, SD of adjusted means and CV in these initial model development steps. Coefficient of variation describes stability, or consistency, in selection of a landscape feature with low values interpreted as depicting consistent use and high values as depicting inconsistent use (*sensu* [65]).

An important assumption of the Cox model is proportionality, meaning that it is assumed that the ratio of estimated hazard functions between individuals will be constant over time. We assessed this assumption by generating time-dependent covariates, which were a product of predictors and survival time, and including them in the model. Statistically significant (p<0.05) time-dependent covariates may indicate non-proportionality [66].

### Mapping the Response and Model Validation

Using the raster calculator tool in Spatial Analyst we created natural log-transformed grids (*i.e.*, for distance variables), as well as grids depicting a quadratic functional form for slope, elevation, and fractional vegetation covariates. For the model of risk of brood failure, we created raster images depicting mean, SD, and CV for covariates using a moving window of 90 m^2^; a window that reflected a spatial scale within which sage-grouse appeared to make patch-level choices (see RSF results and Table 1). We applied the results from resource selection (occurrence) and risk of nest or brood failure models to map relative probability of nest occurrence, resource selection during egg laying/incubation, resource selection during brood-rearing, risk of nest mortality, and risk of brood mortality, respectively, throughout the study area. We used 
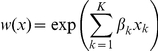
to derive an RSF at a resolution of 30 m where covariates *k* (*k* = 1…*K*) have values *x* [30]. In GIS, we partitioned cells comprising the raster surface into quantiles based on cell value. We reclassified RSF values based on quantiles establishing 5 ranks of the relative probability of occurrence (1 = lowest, 2 = low, 3 = moderate, 4 = high, and 5 = highest). Similarly, we classified relative predicted risk of mortality into the same 5 ranks (1 = lowest to 5 = highest). Using the raster calculator tool we added grids depicting selection of the nest site and selection of resources during incubation and then re-binned the output into 5 equal-sized bins. This output, which gives equal weighting to resources associated with nest-site selection and forays from the nest associated with off-bouts during egg-laying and incubation, depicts the spatial pattern of occurrence during the nesting life-history phase. Using the same method, we combined mid and late brood-rearing occurrence grids to depict the spatial structure of occurrence during the brood-rearing life-history phase. Occurrence during early brood-rearing was not included because this segment of the brood-rearing phase generally mirrors nesting habitat [18].

**Table 1:** Results for models of resource selection during nesting and egg-laying/incubation.

Nest site selection			
Covariate	β	SE	P
Percent bare ground	−0.006	0.064	0.923
Percent bare ground (quadratic)	−0.0003	0.0005	0.632
Percent sagebrush	0.167	0.116	0.151
Percent sagebrush (quadratic)	−0.007	0.005	0.229
Proportion of mesic habitat 810 m	−29.663	12.009	0.014
Distance to nearest road	−0.136	0.056	0.016

Egg-laying and Incubation			
Covariate	β	SE	P
Percent bare ground	0.067	0.028	0.018
Percent bare ground (quadratic)	−0.0008	0.0002	0.002
Percent sagebrush	0.062	0.056	0.276
Percent sagebrush (quadratic)	−0.003	0.002	0.312
Terrain roughness 90 m	−0.245	0.051	<0.001
Distance to mesic habitat	0.293	0.109	0.007
Distance term	7.521	0.622	<0.001
Time of day	0.03	0.004	<0.001
Distance term*time of day	−0.036	0.016	0.024

Table 1. Coefficient estimates (β), associated precision (SE), and significance (P) for models of resource selection during nesting and egg-laying/incubation among sage-grouse that occupy a human-modified landscape in the Intermountain West, USA. As in Table S1, distances (90, 810, and 1590 m) refer to the size of the square moving window within which values were calculated in a GIS.

**Figure 1:**
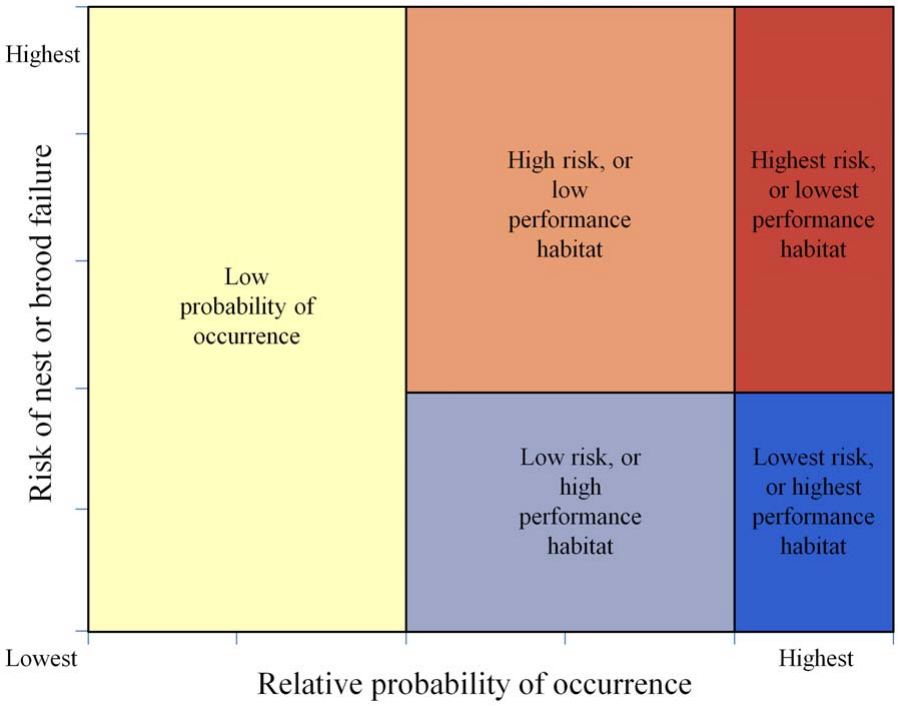
Habitat performance states. Conceptual basis by which habitat performance states were derived.

Next, using Spatial Analyst\Local\Combine, we combined the map of relative probability of occurrence during the nesting life-history phase with the map of risk of nest failure to establish a landscape-level assessment of the relationship between nesting habitat and demographic performance in which 5 habitat states were identified [1]. First (state 1), habitat in which sage-grouse were unlikely to occur was identified. Then (states 2 and 3), habitat in which sage-grouse had a high probability of occurrence coupled with a low risk of nest failure was identified as high-performance. And last (states 4 and 5), habitat in which sage-grouse had a high probability of occurrence coupled with a high risk of nest failure was identified as low-performance (Figure 1). Using the same method, we combined the map of relative probability of occurrence during the brood-rearing life-history phase with the map of risk of brood failure to establish a landscape-level assessment of the relationship between brood-rearing habitat and demographic performance.

The first step in validating spatial models of selection of the nest site, resources selected during egg laying and incubation, and resources selected during brood-rearing included withholding 20% of the sample from RSF development. This meant that models of selection of the nest site and egg-laying/incubation were developed using 70 nests and validated using 18 nests. Models of resources selected during brood-rearing were developed with 18 broods and validated with 4 broods. The validation sample was identified using a random number generator with the constraint that the validation sample for nest site and egg-laying/incubation must be comprised of unique individuals (some individuals re-nested; the 88 nests reflected 68 unique individuals). Each RSF map was validated by plotting the appropriate validation sample on the map and testing whether the number of locations that occurred within each predicted probability of use rank (1–5) differed from expectation using a chi-square test for specified proportions implemented by the FREQ procedure in SAS®.

## Results

This analysis involved 30,207 GPS locations recorded across 68 individual sage-grouse. There were 19,674 locations associated with egg laying and incubation, 5,148 associated with the early brood-rearing period, 3,864 associated with the mid brood-rearing period, and 1,521 with the late brood-rearing period. We analyzed 871 night-time locations during brood-rearing. We monitored 88 nests and 22 broods; there were 64 nest failures and 5 brood failures. Several elements were common among all final resource selection models. Model fit was good with the Wald (Sandwich) test significant in every case (p<0.001). Correlation was high (Pearson product-moment correlation ≥|0.70|) among fractional vegetation covariates so even if several vegetation types were significant we typically were constrained to include ≤2 in any final model. The only interaction term that was significant in some final models was the distance term by time of day interaction; this covariate (as well as the main effects) was included in final models of resource selection during incubation and brood-rearing (Table 1). Last, in several instances the final model of occurrence contains covariates that are not statistically significant at p ≤0.05. This may seem counter-intuitive given component 3 of the covariate inclusion procedure (stepwise variable selection with p ≤0.05 as the criterion for retention). In such cases, the stepwise procedure retained the higher-order term (quadratic) but not the lower-order term (linear). When this was the case, we “forced” the lower-order term into the model, which resulted in slightly adjusted parameter estimates, estimates of precision, and estimates of statistical significance.

### Selection of the Nest Site

In selecting a nest site, sage-grouse avoided areas with a high percentage of bare ground and selected for areas with intermediate values for percentage of sagebrush. The stepwise procedure retained higher-order terms for bare ground and sagebrush. Including the lower-order terms adjusted statistical significance to p>0.05. Nonetheless, we retained these covariates in the model because there is strong biological support for investigating the nesting ecology of sage-grouse relative to shrub vegetation (or lack thereof; see Discussion). Nests also tended to occur closer to roads than expected, and nest placement reflected strong avoidance of areas with a high density of mesic habitat at the 810 m^2^ scale (Table 1).

### Egg-Laying, Incubation, and Brood-Rearing

The final model of resource selection during egg-laying and incubation contained 9 covariates (Table 1). Three of the 9 covariates were the distance term depicting an exponentially decaying probability of use with increasing distance from the nest, time of day, and the interaction between the distance term and time of day. Including distance and time of day terms (and their interaction) improved model fit; AIC with and without these terms was 12,374 and 38,809, respectively.Resource selection during egg laying and incubation was characterized by selection for intermediate levels of both bare ground and sagebrush. Sage-grouse avoided rough terrain at the 90 m^2^ scale, and tended to avoid mesic areas (Table 1).

Although shifts in resource selection patterns were apparent as time progressed from early to late brood-rearing periods (see below), 3 patterns were observed consistently throughout the entire brood-rearing phase. First, sage-grouse consistently showed patch-level (*i.e.*, within 90 m^2^) selection or avoidance of resources. Second, sage-grouse consistently avoided rough terrain at the patch-level. And third, sage-grouse consistently avoided relatively extreme values for vegetation characteristics. During early brood-rearing, sage-grouse selected locally (within 90 m^2^) for an intermediate average percentage of bare ground, showed strong selection locally for habitat in which the average percentage of shrub habitat was high, avoided mesic areas, and tended to select cooler aspects such as north and east-facing slopes. During the mid brood-rearing phase, sage-grouse selected for intermediate levels of shrub habitat and selected locally (within 90 m^2^) for an intermediate average percentage of litter; however, their association with mesic habitat and aspect shifted to selection for mesic areas and for warmer aspects such as south and west-facing slopes, respectively. Also, mid brood-rearing habitat tended to be closer to roads than expected (we tested an interaction term between roads and mesic habitat and, as might be expected given the results of the nest site selection analysis, this interaction was not significant; Table 2). During late brood-rearing sage-grouse selected locally (within 90 m^2^) for intermediate average percentages of bare ground and sagebrush. Sage-grouse showed continued selection for mesic areas and warmer aspects, and for lower elevation than was available locally (Table 2).

**Table 2:** Results for models of resource selection during early, mid, and late brood-rearing.

Early brood rearing			
Covariate	β	SE	P
Average percent bare ground 90 m	0.13	0.049	0.008
Average percent bare ground 90 m (quadratic)	−0.001	0.0004	0.002
Average percent shrub 90 m	0.062	0.019	0.002
Terrain roughness 90 m	−0.165	0.072	0.023
Heat load index	−0.207	0.113	0.068
Distance to mesic habitat	0.104	0.035	0.003
Distance term	3.345	1.011	<0.001
Time of day	.	.	.
Distance term*time of day	−0.017	0.047	0.718

Mid brood rearing			
Covariate	β	SE	P
Percent shrub	0.01	0.047	0.832
Percent shrub (quadratic)	0.001	0.002	0.645
Average percent litter 90 m	0.254	0.111	0.022
Average percent litter 90 m (quadratic)	−0.005	0.002	0.048
Terrain roughness 90 m	−0.125	0.025	<0.001
Heat load index	0.225	0.125	0.073
Distance to mesic habitat	−0.041	0.008	<0.001
Distance to road	−0.05	0.021	0.018
Distance term	6.439	0.918	<0.001
Time of day	.	.	.
Distance term*time of day	−0.245	0.044	<0.001

Late brood rearing			
Covariate	β	SE	P
Average percent bare ground 90 m	0.002	0.042	0.968
Average percent bare ground 90 m (quadratic)	−0.0002	0.0003	0.581
Average percent sagebrush 90 m	0.193	0.113	0.088
Average percent sagebrush 90 m (quadratic)	−0.015	0.007	0.033
Elevation	−0.01	0.002	<0.001
Terrain roughness 90 m	−0.128	0.016	<0.001
Heat load index	0.297	0.216	0.171
Distance to mesic habitat	−0.107	0.033	0.002
Distance term	10.774	1.956	<0.001
Time of day	.	.	.
Distance term*time of day	−0.629	0.121	<0.001

Table 2. Coefficient estimates (β), associated precision (SE), and significance (P) for models of resource selection during early, mid, and late brood-rearing phases among sage-grouse that occupy a human-modified landscape in the Intermountain West, USA. As in Table S1, distances (90, 810, and 1590 m) refer to the size of the square moving window within which values were calculated in a GIS.

### Night-Time Roost Selection

At night during brood-rearing (early, mid, and late phases combined), sage-grouse showed local (within 90 m^2^) avoidance of areas with high average percentage of bare ground. At the scale of larger patches (within 810 m^2^), sage-grouse selected for an intermediate average percentage of sagebrush and avoided rough terrain. Although sage-grouse tended to roost in areas with a high density of mesic habitat at the landscape level (within 1,590 m^2^), they showed avoidance of mesic habitat locally (within 90 m^2^; Table 3).

**Table 3:** Results for the model of resource selection during night-time.

Night-time during brood rearing			
Covariate	β	SE	P
Average percent bare ground 90 m	−0.03	0.011	0.011
Average percent sagebrush 810 m	0.428	0.416	0.303
Average percent sagebrush 810 m (quadratic)	−0.024	0.023	0.308
Terrain roughness 810 m	−0.091	0.023	<0.001
Density of mesic habitat 90 m	−1.827	0.729	0.012
Density of mesic habitat 1590 m	9.632	4.896	0.049
Distance term	107.616	21.971	<0.001
Time of day	.	.	.
Distance term*time of day	−5.1	1.036	<0.001

Table 3. Coefficient estimates (β), associated precision (SE), and significance (P) for the model of resource selection at night during the brood-rearing phase among sage-grouse that occupy a human-modified landscape in the Intermountain West, USA. As in Table S1, distances (90, 810, and 1590 m) refer to the size of the square moving window within which values were calculated in a GIS.

### Risk of the Nest and Brood Failure

The final nest risk model fit the data well (Wald Sandwich test χ^2^ = 31.83, df = 4, p<0.001) and included 4 covariates (Table 4). No time-dependent covariate was statistically significant (in nest or brood risk models) suggesting the assumption of proportionality was met. Risk of nest failure was driven by the percentage of bare ground at the nest location, proximity to mesic areas, and proximity to natural gas or oil wells that existed or were installed the previous year (a 1-year time lag). The higher the percentage of bare ground comprising the grid cell in which the nest was located, the more likely it was to fail. The closer a nest was to mesic habitat or to wells (that existed of were installed in the previous year), the more likely it was to fail. The final brood risk model also fit the data well (Wald Sandwich test χ^2^ = 27.98, df = 3, p<0.001) and included 3 covariates that depicted mean, SD, and CV values across brood locations within each brooded female sage-grouse (Table 4). The farther a brooded female was (on average) from mesic habitat, the more likely it was for that female's brood to fail. Risk of brood failure was high when variability in selection for slope by the brooded female was high (SD of slope). Last, risk of brood failure was high when the brooded female showed stable or consistent selection for lower elevation (CV of elevation; Table 4) as revealed by plots of mean ± SD and CV.

**Table 4:** Results for Cox proportional hazards models of risk of nest and brood failure.

Risk of nest failure					
Covariate	β	SE	P	Hazardratio	95% hazard ratio confidence limits
Percent bare ground	0.077	0.051	0.136	1.08	0.976–1.196
Percent bare ground (quadratic)	−0.0007	0.0004	0.088	0.999	0.998–1.0
Distance to mesic habitat	−0.238	0.043	<0.001	0.788	0.723–0.858
Distance to nearest well (1 yr time lag)	−0.423	0.175	0.016	0.655	0.464–0.925

Risk of brood failure					
Covariate	β	SE	P	Hazardratio^a^	95% hazard ratio confidence limits
Standard deviation (SD) of slope	2.609	1.418	0.066	13.587	0.844–218.818
Coefficient of variation (CV) of elevation	−21.66	4.973	<0.001	0.119^a^	0.045–0.316
Mean distance to mesic habitat	0.143	0.022	<0.001	1.159	1.111–1.210

Table 4. Coefficient estimates (β), precision (SE), significance (P), and estimated hazard ratios and corresponding confidence limits (CL) for the Cox proportional hazards model of risk of nest and brood failure among sage-grouse that occupy a human-modified landscape in the Intermountain West, USA.

a Hazard ratio calculated based on a unit of 1 m, 0.1 units, and 1 m for SD of slope, CV of elevation, and mean distance to mesic habitat, respectively.

### Mapping the Response and Model Validation

**Figure 2:**
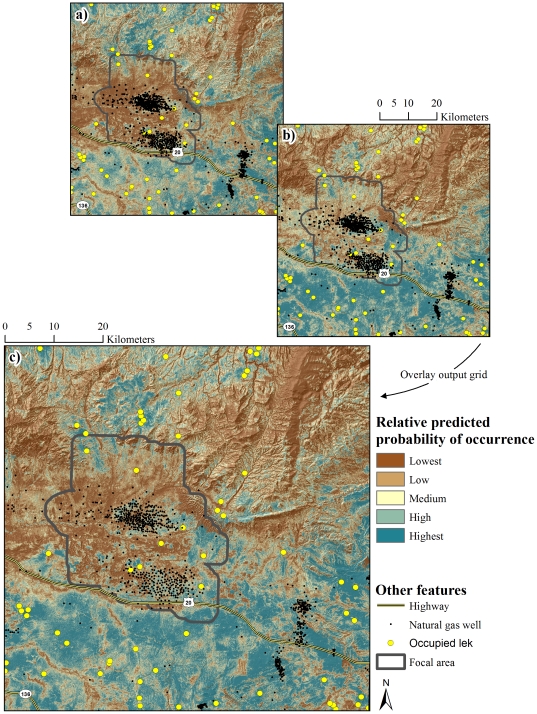
Occurrence during the nesting life-history phase. Relative predicted probability of nest-site selection (a), and occurrence during egg-laying and incubation (b). Panels (a) and (b) were combined generating a depiction of relative predicted probability of occurrence during the nesting life-history phase (c).

**Figure 3:**
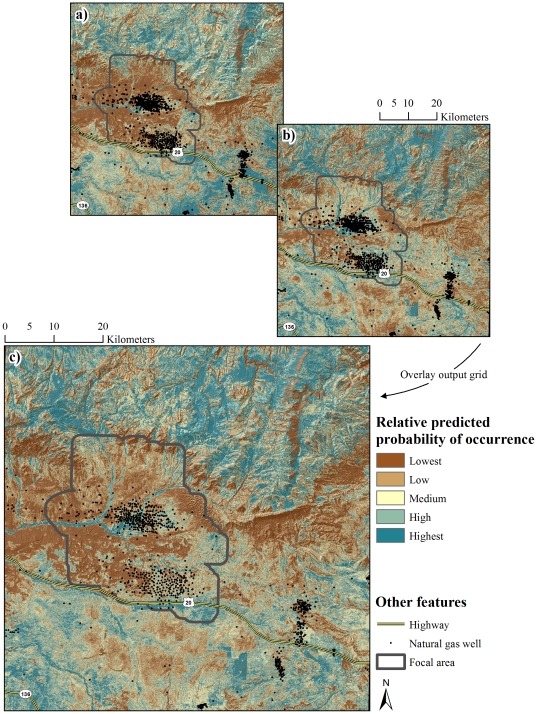
Occurrence during the brood rearing life-history phase. Relative predicted probability of occurrence during mid brood rearing (a) and late brood rearing (b). Panels (a) and (b) were combined generating a depiction of relative predicted probability of occurrence during the brood rearing life-history phase (c).

**Figure 4:**
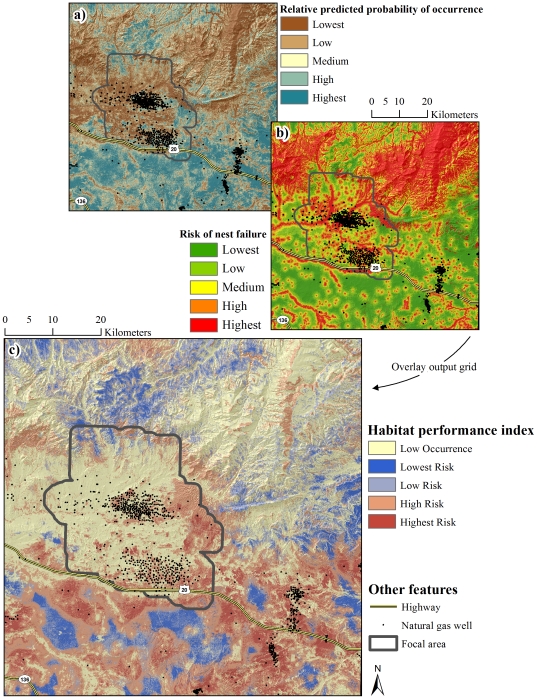
Nesting habitat performance index. Relative predicted probability of occurrence during the nesting life-history phase (a) and risk of nest failure (b) were combined to generate a nesting habitat performance index (c).

**Figure 5:**
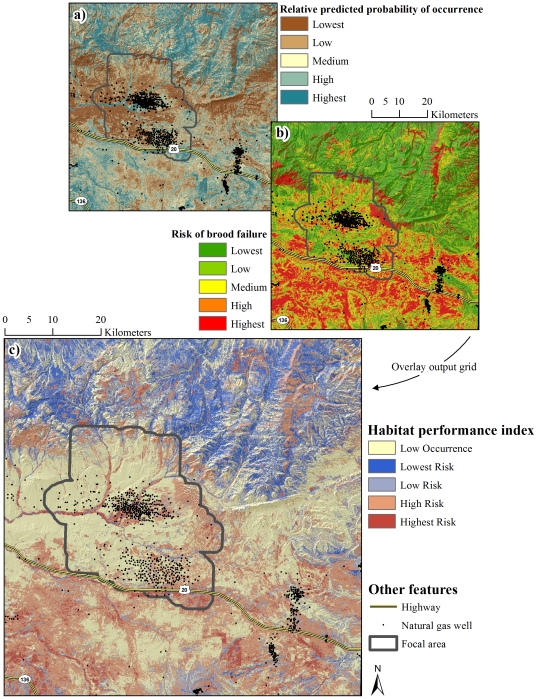
Brood rearing habitat performance index. Relative predicted probability of occurrence during the brood rearing life-history phase (a) and risk of brood failure (b) were combined to generate a brood rearing habitat performance index (c).

**Figure 6:**
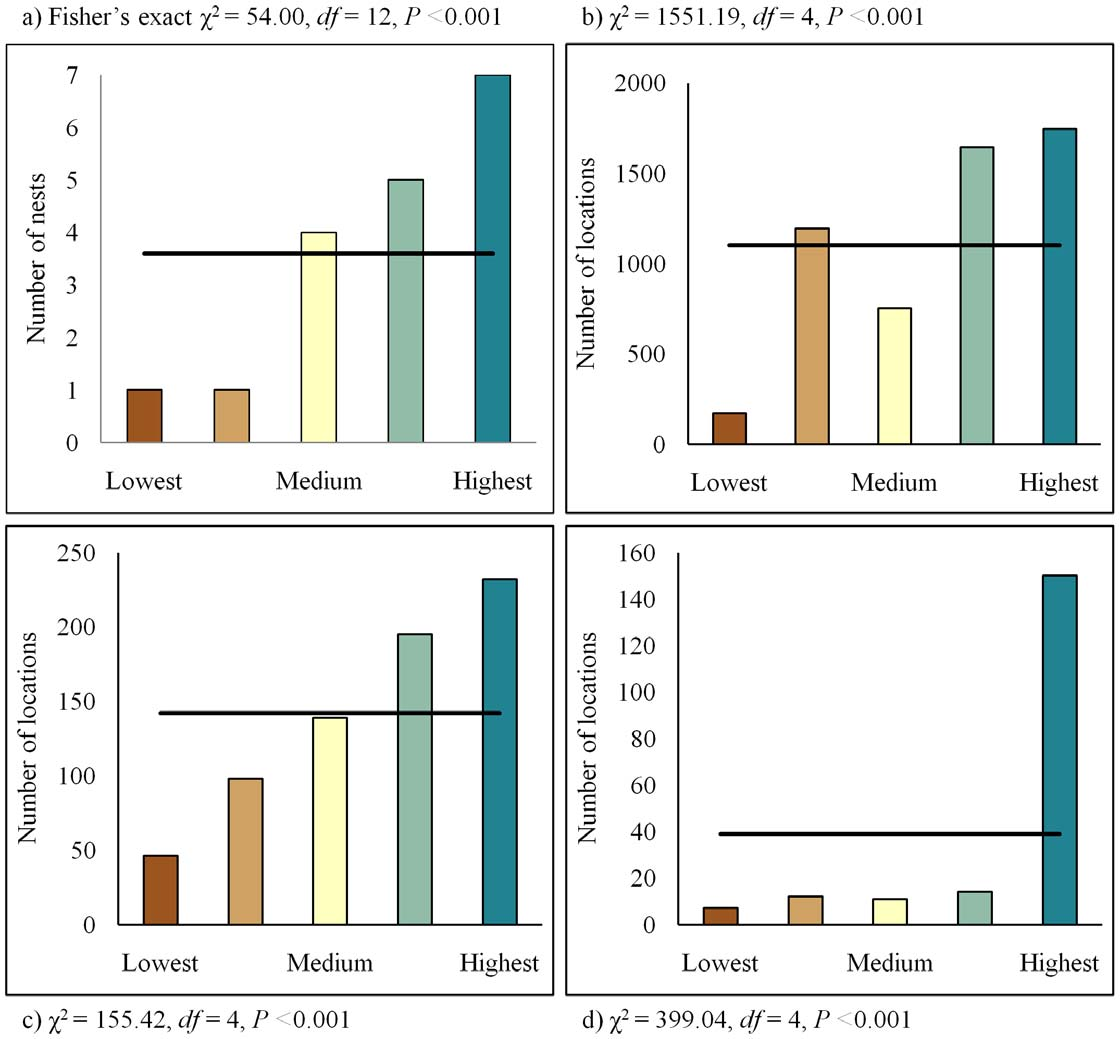
Model validation. Validation of nest occurrence (a), egg-laying/incubation occurrence (b), mid brood rearing occurrence (c), and late brood rearing occurrence (d) models.

Maps of relative predicted probability of occurrence were developed for nest occurrence, occurrence during egg-laying and incubation, and occurrence during mid and late brood rearing (Figures 2 and 3). Separate maps of relative risk of nest failure (Figure 4b) and brood failure (Figure 5b) were likewise developed. Nesting, egg-laying/incubation, early, mid, and late brood-rearing were validated with 18, 5,504, 1,025, 710, and 194 locations, respectively. All predicted occurrence maps (Figures 2 and 3) validated well with far fewer validation locations occurring in low predicted probability of occurrence bins, and far more validation locations occurring in high predicted probability of occurrence bins relative to that which would be expected by chance (Figure 6).

Considering the nesting habitat performance index (Figure 4c), we identified 55.9% of the landscape as having a medium to high relative probability of nest occurrence. High risk habitat, where high probability of occurrence coincided with high risk of nest mortality, was identified throughout 33.3% of the landscape, with 6.9% of the landscape characterized as highest risk (Figure 4c). High probability of occurrence coincided with a low risk of nest mortality throughout 22.8% of the landscape, with 6.6% of the landscape characterized as lowest risk (Figure 4c). Examining the brood-rearing habitat performance index (Figure 5c), 59.7% of the landscape had a medium to high relative probability of brood occurrence. High risk habitat was identified throughout 29.6% of the landscape, with 4.3% characterized as highest risk. Low risk habitat was identified throughout 30.1% of the landscape, with 10.8% of the landscape characterized as lowest risk (Figure 5c).

## Discussion

### Habitat-Performance Relationships and GPS Data

Spatial models that link habitat with metrics of demographic performance in animals have strong application in conservation planning because they identify and depict the landscape in terms of its likely function in population persistence, and the analysis from which spatial predictions were derived provides information on mechanisms driving observed spatial patterns [1], [2], [67]. Spatial modeling for animal conservation typically does not include predictions of demographic performance; rather, patterns of occurrence are relied upon for guidance of conservation action (*sensu* [12]). Relying on animal occurrence alone risks focusing effort and funding on an area that functions as attractive sink habitat where occurrence may be high yet demographic performance is poor [5], [29]. Alternatively, spatial patterns of occurrence in human-modified landscapes may reflect risk aversive behavior in animals (*i.e.*, human-modified behavior; [12]). In this situation, occurrence may be low in places that otherwise would confer high fitness [68]. While the conceptual framework of linking occurrence and fitness for animal conservation has general application across taxa and landscape types, the mapping tools developed herein will have specific application in guiding on-the-ground conservation activity relative to high quality nesting and brood-rearing habitat throughout the study area.

This work offers the first reported use of GPS telemetry on North American prairie grouse in peer-reviewed published literature (*see* [69] for the only documentation of GPS use in Galliformes). The volume and precision of GPS data acquired in this work allowed the specific research questions, rather than data availability or quality, to determine the most appropriate analytical framework. The discrete-choice approach accounts for biological processes underpinning behavior in gallinaceous birds perhaps to a greater extent than unconditional analytical methods to which investigators have been bound by conventional telemetry data, particularly during reproductive phases when nests or broods impose constraints on movement and thus habitat availability [41]. Further, GPS opened the door to investigating facets of sage-grouse ecology that have been difficult to document such as resource selection during egg-laying, incubation, and night-time roosting.

### Occurrence during Reproductive Phases

In this central Wyoming landscape, the choice of where to situate a nest reflected avoidance of bare ground and mesic areas, and selection for moderate sagebrush cover. Previous research also documented landscape-level avoidance of bare ground and selection for moderate shrub cover [2], [70]. Resources selected during forays from the nest as part of egg-laying and incubation behavior showed some distinction from the nest site itself. Specifically, sage-grouse showed marked avoidance of rugged terrain during forays and were more likely to occur in places with moderate levels of bare ground. Incubation behavior in birds, particularly among species in which only females incubate, can be viewed as a trade-off between self-maintenance by the female and care of the (un-hatched) young. This trade-off is mediated by the frequency and length of on- and off-bouts; [71] and includes features such as maintaining energy requirements of the female, thermal requirements of the developing embryos, and the need to minimize activity that could attract visually-oriented predators [71], [72]. In our study, forays from the nest that provided the basis for documenting resource selection during incubation reflected such off-bouts which likely were related to foraging (maintenance of energy requirements of the female [73], [74]). Spatial modeling of resources that are important during the nesting phase in birds typically does not include prediction of occurrence during incubation. However, resources selected by incubating females on forays can be viewed as a critical component of the nesting phase (Figure 2b). Incorporating spatial prediction of occurrence during incubation, in addition to predictions of nest-site selection, enhances the practical utility of RSF mapping tools by accounting for a more complete range of the biological needs that govern occurrence in nesting birds.

Patch-level selection characterized resource-related choices made by brooded females. Small patches (90 m^2^ or 0.8 ha) with moderate levels of bare ground, litter, and shrub (particularly sagebrush during late brood-rearing) were selected throughout brood-rearing. It is thought that patchy cover offers the necessary forb resources while providing refugia from predation [2]. Females showed an aversion to mesic areas during early brood-rearing (similar to nest-site selection) but strong selection for wet areas during mid and late phases. Mesic areas provide forbs and invertebrates (critical components of the diet) and become increasingly important as other sagebrush habitats desiccate in June and July. Generally, features of brood-rearing habitat for sage-grouse have become well known, with 4 patterns emerging consistently. First, while some research has indicated proportional use or avoidance of shrub coverage [75], [76], most research has shown that shrub cover is a key component of brood-rearing habitat [2], [77], [78], [79], [80]. Second, brooded females tend to select for higher proportions of litter or herbaceous cover that provides habitat for important food sources such as invertebrates and forbs [75], [76] (*but see* [80]). Third, mesic habitat plays a critical role in structuring the occurrence of brooded females, with females generally showing strong selection for such habitat [2], [78], [81]. And fourth, regional variation in local-scale habitat attributes, particularly in the density and size of shrubs as well as the association between shrub coverage and understory composition, is a key consideration in interpreting differences among studies [79], [82].

We found that several features of night-time occurrence mirrored day-time occurrence, such as selection for moderate sagebrush cover and avoidance of rough terrain. However, at night the association between occurrence and mesic habitat reflected choices made locally and at the landscape level with females selecting strongly for landscapes (within 1,590 m^2^ or 625 ha) with a high density of mesic habitat, but avoiding mesic habitat locally (within 90 m^2^ or 0.8 ha). Others [83] have described night roost habitat (among prairie chickens [*Tympanuchus* spp.]) as a process involving perception of resources at multiple spatial scales, with sparse cover characterizing the roost site locally and tall dense vegetation characterizing the roost site at a larger spatial scale. In our study area, brooded females roosted in landscapes that offered access to key day-time foraging habitat but, within such landscapes, avoided direct association with mesic areas as a likely mechanism to reduce risk of predation [75], [76], [80], [81].

### Risk of Nest and Brood Failure

Risk of nest failure in prairie grouse is tied primarily to predation [34], [35], [36], [37], [38]. In this study, 60 of 64 nest failures were attributed to predation. Bare ground in the immediate vicinity of the nest location, even at moderate levels, increased risk of nest failure as did proximity to natural gas or oil wells that had been drilled no later than the previous year. These results are consistent with previous observations; low shrub canopy coverage at the nest site has been linked with nest failure [38], [84], [85]. Likewise, a time-lag in the influence of human activity on sage-grouse reproductive activity has been documented [21].

**Figure 7:**
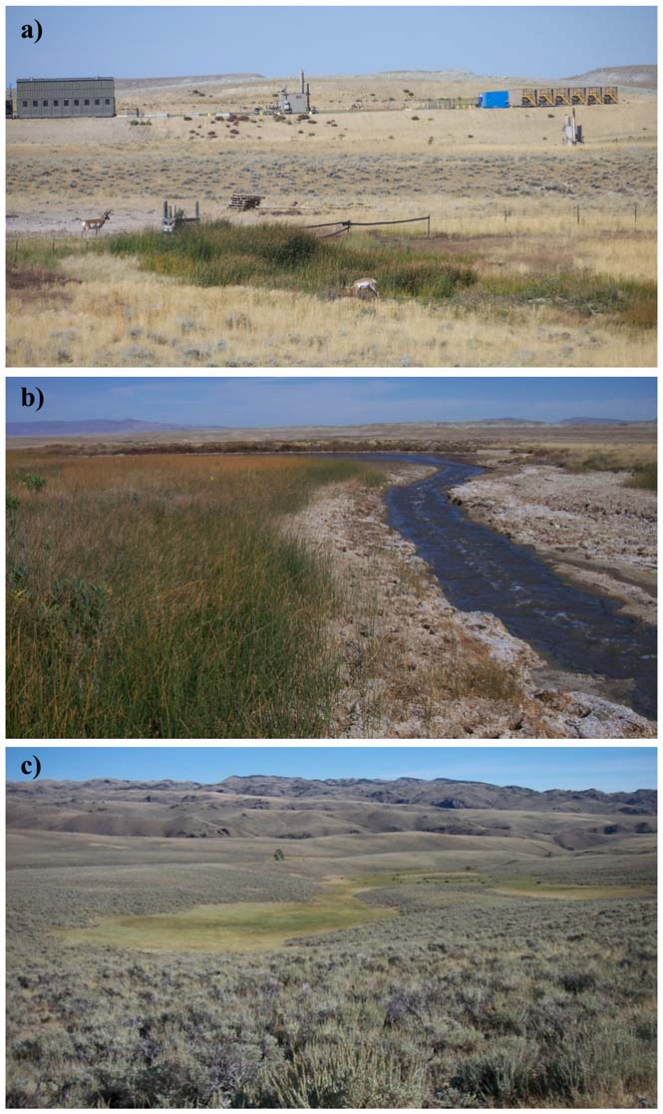
Anthropogenic versus natural water features. Anthropogenic water features at low elevation on the study area including an agricultural water development (a) and discharge area for water produced from energy development (b), contrasted with a natural water feature at high elevation where snowmelt pools and mesic conditions persist throughout summer (c). Photos by C.V. Olson.

Proximity to mesic habitat strongly increased risk of nest failure. Implications of this finding may be far-reaching depending on whether energy development is a feature of the landscape, and whether management of water produced as a byproduct of such development is an issue, as is the case in this study area and other landscapes where industrial development is occurring or planned. Throughout the Intermountain West, the spatial configuration of water in arid, lower-elevation basins is largely a function of human activity associated with agriculture and, more recently, energy development. This is in contrast to higher-elevation wet areas which generally are natural features of the landscape. Our study site exemplifies this contrast; wet areas in lower-elevation portions of the site were nearly entirely anthropogenic features (Figure 7a and b) whereas wet areas at higher-elevation were natural landscape features (Figure 7c). Nest survival in grassland-nesting ducks (*Anas* and *Aythya* spp.) was negatively related to the surrounding density of mesic habitat [86]. They [86] noted that several species of nest predators (*e.g.*, *Mephitis mephitis*) preferentially select mesic habitat for foraging and they speculated that the higher productivity of mesic areas supported a greater number of predators.

Proximity to mesic habitat decreased risk of brood failure. However, it appears that the relationship between risk and mesic habitat was mediated by elevation. All broods that failed showed consistency in use of lower-elevation, as revealed by the metric CV of elevation. This pattern may reflect two general processes. It is possible that consistent or predictable use of a resource (*i.e.*, low values for CV) reflects an inability to adjust to complex environmental conditions, whereas less-consistent use of a resource (*i.e.*, higher values for CV) reflects a form of plasticity through which individuals are more successfully able to navigate and respond to complexities in their environment [87]. Alternatively, the possible link among mesic habitat, lower elevation, and risk of brood failure could reflect shorter duration under observation among failed broods. All broods that failed did so within 15 days of leaving the nest. Including measures of variation in models of risk or occurrence [2], [86] can provide insight into patterns that are not readily discernable from average responses, particularly in human-modified landscapes where human activity can effect subtle influences on the functional properties of a landscape feature (e.g., mesic habitat).

### Sage-Grouse Conservation in Human-Modified Landscapes

Findings of this investigation have their strongest application in establishing a spatially-explicit basis from which to minimize risk of nest and brood failure. Variability in the spatial structure of occurrence of sage-grouse was driven largely by selection or avoidance of terrain and vegetation features. In contrast, risk of nest and brood failure was largely a function of human modification of the landscape with relatively little variation explained by habitat, particularly when it is recognized that mesic habitat in arid, lower-elevation portions of the landscape were anthropogenic features. This apparent bottom-up, top-down pattern of influence on occurrence and risk, respectively, has been noted previously [67] and establishes as necessary an approach to conservation planning for sage-grouse in which management of human activity at the landscape level is prioritized over local-scale habitat improvement projects. The issue of water produced as a byproduct of energy development offers an example of the applied value of RSF maps (Figures 4 and 5) in guiding management of human activity in the interest of population persistence.

**Figure 8:**
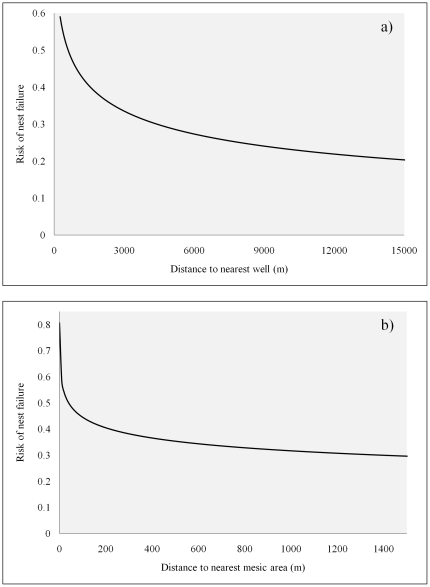
Nest failure as a function of anthropogenic features. Resource selection curves depicting risk of nest failure as a function of proximity to the nearest oil or natural gas well (a) and mesic area (b). We generated selection curves by calculating the inverse logit of the linear model, inputting different values for distance to nearest well or mesic area while holding all covariates constant at their mean values.

The use of produced water to create mesic habitat such as wetlands, wet meadows, or impoundments has been suggested as a method for improving or creating habitat for sage-grouse [79], [88]. Such water improvements offer a mechanism for managing produced water while establishing a habitat type that brooded females are known to select [2], [78], [81]. Using produced water to create habitat conditions known to promote occurrence during brood-rearing appears to risk fewer broods present on the landscape through deleterious effects on nest success, particularly when water improvements are situated in habitat where sage-grouse are likely to nest. Likewise during brood-rearing, consistent use of low elevation mesic (anthropogenic features) habitat was associated with a higher rate of brood failure. If management of produced water is an issue, water discharge or development of water improvements should be conducted in areas with a low probability of nest and early brood occurrence. The area predicted to have low occurrence encompassed 44.1% and 40.3% of the landscape for nests and broods, respectively (Figures 4c, 5c); these low-occurrence areas likewise provide the basis for the notion that constraints on human activity should be focused in specific areas (see below) rather than enacted region-wide. As a function of distance to the nearest well, risk of nest failure at ∼1,600 m from a well was two-thirds that of nests ∼250 m from a well (Figure 8a). Similarly, risk of nest failure at ∼200 m from the nearest mesic area was half that of nests ∼1 m from a mesic area (Figure 8b). Industry and the managing agencies could apply these results by aiming for a specific-percent reduction in risk of nest failure as part of new construction by constraining infrastructure or water management activity within a given distance of high-probability-of-occurrence nesting habitat (55.9% of the study area; Figures 4a, c). For example, constraining water management discharge to areas ≥200 m from nesting habitat is predicted to reduce absolute risk of nest failure by 50% (Figure 8). Constraining new well development to areas ≥1,600 m from nesting habitat is predicted to reduce absolute risk of nest failure by 33% (Figure 8). Maps of nesting and brood-rearing habitat-performance indices (Figures 4c, 5c) offer a tool for refining management plans in ways that have not been feasible using maps of occurrence alone. For example, spatial depiction of high-performance habitat (22.8% and 30.1% of the landscape for nesting and brood-rearing, respectively) where sage-grouse are likely to occur and have high nest or brood success should be viewed as explicit identification and prioritization of habitat in which further human-modification should be minimized to the greatest extent feasible (Figures 4c, 5c). Spatial depiction of low-performance habitat (33.3% and 29.6% of the landscape for nesting and brood-rearing, respectively) where sage-grouse are likely to occur but have low nest or brood success (*i.e.*, sink habitat) should be viewed as explicit identification and prioritization of habitat for which industry and the managing agencies ask: considering these maps (Figures 4c, 5c), what can we do to change red to blue? We offer answers to this question within the context of hypothesizing as to why human-modification of the landscape increases risk to sage-grouse.

The spatial pattern of risk during nesting and brood-rearing suggested a human-mediated increase in predator abundance or effectiveness as one potential mechanism of landscape-level variation in nest and brood success [9], [89]. Infrastructure associated with industrial development offers a relatively high density of habitat for predators including culverts, pipe yards, buildings, and storage facilities. These features likewise offer refugia for many small mammal species that provide a prey base for larger predators. Some industrial facilities such as storage tanks, communication towers, and buildings provide nesting substrate for avian predators. Industrial development may enhance the effectiveness of visually-oriented avian predators by providing structures such as utility poles that can be used as perches. It is likely that anthropogenic mesic habitat, particularly in and adjacent to industrial areas, functions to concentrate animal (prey) activity in spatially and temporally predictable ways further enhancing predator effectiveness. Such subsidization of generalist predators can have severe impacts to populations of sensitive prey species because subsidies decouple predator populations from declines in specific prey species [10]. Although the notion of a “human-mediated increase in predator abundance or effectiveness” is a hypothesis that requires further testing, we move forward with management recommendations that address predation because data analyzed herein show that predation was the primary driver of nest and brood failure, and risk of failure was greater in and around human-modified areas.

We note that the issue of predator subsidization is readily amenable to management intervention and, as such, we can begin to address the question posed above about changing red to blue (Figures 4c, 5c). In low-performance habitat, restricting access by potential predators to refugia (*i.e.*, fencing culverts), burying utility lines, removing utility poles, and discouraging the use of facilities as nesting substrate for avian predators would reduce predator density and effectiveness (*sensu* [90]). If feasible, eliminating water developments in low-performance habitat, or consolidating such development, will likely reduce predator effectiveness. Considering elevational movement exhibited by sage-grouse in this study area, anthropogenic risk factors operated primarily during nesting and early brood-rearing phases before many sage-grouse moved up in elevation. Habitat selected by nesting and incubating sage-grouse closely mirrored habitat selected during early brood-rearing. Implicit in recommendations to avoid or minimize the creation of new anthropogenic risk factors in high-performance habitat and to take steps to reduce existing risk factors that render a habitat low-performance is the idea that nest success is perhaps the most important demographic metric governing population change in human-modified landscapes. It is with this in mind that we recommend a strong focus on better understanding mechanisms driving the observed pattern of risk during nesting (*i.e.*, human-mediated predator subsidization), and on leveraging spatial tools (Figures 4 and 5) for identifying and prioritizing where conservation intervention is expected to be most effective.

## Supporting Information

Table S1 **Covariates calculated in a Geographic Information System (GIS; ArcMap 9.3) for potential inclusion in models of resource selection and risk of nest and brood failure among greater sage-grouse.** A description of each covariate is provided in the right-hand column. All data (raster images) were calculated at a resolution of 30 m. Also shown is conversion among units-of-area for the spatial scales at which covariates were calculated.
(DOC)
