# Supplementary material for: Identifying and Prioritizing Greater Sage-Grouse Nesting and Brood-Rearing Habitat for Conservation in Human-Modified Landscapes
Source: PLoS One. 2011 Oct 13;6(10):e26273. doi: 10.1371/journal.pone.0026273 (PMC3192796; doi:10.1371/journal.pone.0026273)
Supplement: Table S1 — Covariates calculated in a Geographic Information System (GIS; ArcMap 9.3) for potential inclusion in models of resource selection and risk of nest and brood failure among greater sage-grouse. A description of each covariate is provided in the right-hand column. All data (raster images) were calculated at a resolution of 30 m. Also shown is conversion among units-of-area for the spatial scales at which covariates were calculated. (DOC) [file pone.0026273.s001.doc]

Table S1. Covariates considered in resource selection and risk models.

| **Predominant human modifications of the landscape** | | | | | |
| --- | --- | --- | --- | --- | --- |
| Distance to nearest well | | Distance (m) to the nearest oil or natural gas well. Data on the location of wells, as of July 2010, were obtained from the Wyoming Oil and Gas Conservation Commission. For the nest occurrence model, we evaluated this covariate as a 2-, 1-, or 0-year time-lag. All distance covariates were calculated using the Spatial Analyst\Euclidean Distance tool in ArcMap. | | | |
| Distance to nearest road | | Distance (m) to paved, improved un-paved, or maintained dirt roads. Two-track roads were not included. Roads were heads-up-digitized at a scale of 1:2000 from 2006 and 2009 1-m resolution National Agriculture Imagery Program aerial imagery. | | | |
| Distance to nearest residential or agricultural structure | | Distance (m) to the nearest residential or agricultural structure including houses, sheds, and barns. Structures were heads-up-digitized as described above. | | | |
| Distance to nearest energy-related ancillary feature | | Distance (m) to the nearest infrastructure associated with energy development other than wells. Such ancillary features included compressor stations, settling ponds, and buildings. Ancillary features were heads-up-digitized as described above. | | | |
| **Predominant vegetation in the study area** | | | | | |
| Percent shrub | | Estimated percent of each pixel comprising the raster surface for which the vegetation type is shrub species. The data source was the Provisional Remote Sensing Sagebrush Habitat Quantification Products for Wyoming developed by the U.S. Geological Survey. Detailed information on development and accuracy of all vegetation layers is in [27]. | | | |
| Percent sagebrush | | Estimated percent of each pixel comprising the raster surface for which the vegetation type is sagebrush (*Artemisia* spp.). | | | |
| Percent bare ground | | Estimated percent of each pixel comprising the raster surface that is bare ground. | | | |
| Percent herbaceous cover | | Estimated percent of each pixel comprising the raster surface that is herbaceous vegetation. | | | |
| Percent litter | | Estimated percent of each pixel comprising the raster surface that is herbaceous litter. | | | |
| Average percent shrub 90 m, 810 m, 1590 m | | Average percent shrub calculated for each pixel within moving windows of 90, 810, and 1590 m on a side. | | | |
| Average percent bare ground 90 m, 810 m, 1590 m | | Average percent bare ground calculated for each pixel within moving windows of 90, 810, and 1590 m on a side. | | | |
| Average percent herbaceous cover 90 m, 810 m, 1590 m | | Average percent herbaceous cover calculated for each pixel within moving windows of 90, 810, and 1590 m on a side. | | | |
| Average percent litter 90 m, 810 m, 1590 m | | Average percent litter calculated for each pixel within moving windows of 90, 810, and 1590 m on a side. | | | |
| **Topographic and other natural features of the landscape** | | | | | |
| Elevation | | Elevation (m) calculated from a digital elevation model (DEM). | | | |
| Heat load index (HLI) | | Rescaling of aspect (*θ*; radians) from 0 – 1 oriented northeast to southwest depicting the gradient from coolest to warmest aspect using the equation of [91]: HLI = 1 – cosine(*θ* – 45)/2 | | | |
| Slope | | Steepness (degrees) calculated from a DEM. | | | |
| Terrain roughness 90 m, 810 m, 1590 m | | An index of terrain roughness calculated as the standard deviation (SD) of elevations from a DEM within a moving window of 90, 810, and 1590 m on a side. These covariates were calculated using the Spatial Analyst\Local\Cell Statistics tool selecting SD as the overlay statistic. | | | |
| Distance to nearest mesic area | | Distance (m) to nearest permanent or intermittent stream, seep, spring, impoundment, irrigation, or water discharge area. The raster image was developed from 0.3-m true-color and CIR aerial photography (2009) using Feature Analyst® 4.2 [92] for ArcGIS® 9.3. Readers should consult [67] for details on image classification using Feature Analyst®. | | | |
| Proportion of mesic habitat (as above) 90 m, 810 m, 1590 m | | Proportion of the area within a moving window of 90, 810, and 1590 m on a side that is comprised of mesic habitat. These covariates were calculated using the Spatial Analyst\Neighborhood\Focal Statistics tool with sum specified as the statistic type (which provides the sum total of pixels representing the habitat of interest within the moving window), and later converted to proportion of area (km^2^/km^2^). | | | |
| **Scale conversion** | | | | | |
| Moving window | Number of cells | Km^2^ | Hectares | Acres | Miles^2^ |
| 90 × 90 m | 3 × 3 | 0.008 | 0.8 | 1.98 | 0.003 |
| 810 × 810 m | 27 × 27 | 0.656 | 65.6 | 162.1 | 0.253 |
| 1590 × 1590 m | 53 × 53 | 2.528 | 252.8 | 624.7 | 0.976 |

Table S1. Covariates calculated in a Geographic Information System (GIS; ArcMap 9.3) for potential inclusion in models of resource selection and risk of nest and brood failure among greater sage-grouse. A description of each covariate is provided in the right-hand column. All data (raster images) were calculated at a resolution of 30 m. Also shown is conversion among units-of-area for the spatial scales at which covariates were calculated.
